# Pre-Operative, High-IL-6 Blood Level is a Risk Factor of Post-Operative Delirium Onset in Old Patients

**DOI:** 10.3389/fendo.2014.00173

**Published:** 2014-10-17

**Authors:** Miriam Capri, Stella Lukas Yani, Rabih Chattat, Daniela Fortuna, Laura Bucci, Catia Lanzarini, Cristina Morsiani, Fausto Catena, Luca Ansaloni, Marco Adversi, Maria Rita Melotti, Gianfranco Di Nino, Claudio Franceschi

**Affiliations:** ^1^Department of Experimental, Diagnostic and Specialty Medicine, University of Bologna, Bologna, Italy; ^2^Interdepartmental Center Galvani, University of Bologna, Bologna, Italy; ^3^Institute for Biomedical Aging Research, University of Innsbruck, Innsbruck, Austria; ^4^Department of Psychology, University of Bologna, Bologna, Italy; ^5^Agency for Health and Social Care of Emilia-Romagna, Bologna, Italy; ^6^Units of General, Emergency and Transplant Surgery, St. Orsola-Malpighi Hospital, Bologna, Italy; ^7^General Surgery I, Ospedali Riuniti di Bergamo, Bergamo, Italy; ^8^Department of Surgical and Anesthesiological Sciences, St. Orsola-Malpighi Hospital, Bologna, Italy

**Keywords:** post-operative delirium, IL-6, inflammatory cytokines, aging, inflammaging

## Abstract

**Background:** Post-operative delirium (POD) is a common complication in elderly patients undergoing surgery, but the underpinning causes are not clear. We hypothesized that *inflammaging*, the subclinical low and chronic grade inflammation characteristic of old people, can contribute to POD onset. Accordingly, we investigated the association of pre-operative and circulating cytokines in elderly patients (>65 years), admitted for elective and emergency surgery.

**Methods:** This is a secondary analysis of a sub-cohort of patients belonging to a previous large case–control study, where 351 patients were clinically and cognitively thoroughly characterized, together with the assessment of POD (47 patients) by confusion assessment method and delirium rating scale. Seventy-four pre-operative plasma samples were selected from a larger bio-bank and they included 37 subjects with POD and 37 without POD. Inflammaging related cytokines, i.e., IL-1β, IL-2, IL-6, IL-8, IL-10, and TNF-α, were assayed by ELISA in pre-operative blood samples; univariate and multivariable analyses have been applied to identify cytokines independently associated to POD. Associations of cytokine levels with functional status, cognitive decline, intra-hospital mortality, and comorbidity were also analyzed independently of POD onset.

**Results:** High IL-6 and low-IL-2 levels were significantly associated with POD. After adjustment for potential confounders in multivariate analysis, high level of pre-operative IL-6 was confirmed to be significantly associated with risk of POD onset. High level of IL-6 was also associated with several baseline features (including poor functional status, cognitive impairment, emergency admission, and higher comorbidity burden) and intra-hospital mortality.

**Conclusion:** Pre-operative, high-plasma level of IL-6 (≥9 pg/mL) was significantly associated with POD onset. We propose IL-6 as an additional risk factor of POD onset together with the previously identified factors. Discovery of all risk factors contributing to POD onset will permit to improve hospitalized patient management and the decrease of healthcare cost.

## Introduction

Postoperative delirium (POD) is a common complication predominantly in old patients undergoing major surgical procedures with heavy effects in terms of hospital costs (1). It is associated with poor outcomes, such as prolonged hospital stays, increased risk of death, functional and cognitive decline, and a higher rate of institutionalization. It is well established that delirium incidence differs in dependence of many factors, among them, the clinical setting has a prominent role: frequency ranges from 11 to 42% in the case of medical patients and can reach higher rates in elderly after hip fracture (2).

Recently, Inouye indicated the contribution of several predisposing and precipitating risk factors and their possible combined actions in delirium onset (3). Despite an increase of published articles focused on the identifications of delirium risk factors, the molecular mechanisms are only partly understood (4) and notably, the possibility for early identification of subjects who may develop POD is still to be better defined. Different theories were proposed to explain delirium pathogenesis; among these, the hypotheses of neurotransmitters imbalance (5) and inflammatory impairment (6) are the most widely propagated. Our working hypothesis is that neuroinflammation leads to POD development as recently suggested by different authors (7, 8).

It is established that human aging is associated with a chronic low-level pro-inflammatory status, named by our group *inflammaging* (9, 10) to which the age-related remodeling of the immune system greatly contributes. Inflammaging has both local and systemic effects and this status may favor, especially when other predisposing factors are concomitant, an increased risk of age-related diseases onset (11, 12). The unbalance of pro- and anti-inflammatory cytokine plasma levels could be a predisposing factor of POD onset in the pre-operative status of hospital admitted elderly patients.

In a previous work, we identified a protocol for a first feasibility study (13) taking into account the hospital unit setting of enrolled patients (Unit of General, Emergency, and Transplant Surgery; St. Orsola-Malpighi Hospital, Bologna, Italy). Subsequently, in a second work, different variables were evaluated and some risk factors were identified as follows: (i) age ≥75 years; (ii) cumulative illness rating scale (CIRS) ≥8, related to comorbidity; (iii) hospital anxiety depression scale (HADS) ≥15, associated with depression; (iv) short-portable mental state questionnaire (SPMSQ) ≤7, associated with cognitive decline; (v) deregulated pre-operative glycemia (14). A large database containing 351 enrolled patients (out of 1,235 subjects assessed for eligibility) in a case–control study was realized. The present study aimed to further investigate predictive factors of POD assessing pre-operative-inflammaging related-cytokines plasma level.

TNF-α, IL-1beta, IL-2, IL-6, IL-8, and IL-10 cytokines were determined as secondary analysis in a subgroup of old patients who were admitted for elective and emergence surgery, as described previously (14). Furthermore, we also investigated the association of the above mentioned cytokines independently of POD onset with age ≥75 years, CIRS ≥8, HADS ≥15, SPMSQ ≤7, emergence surgery, and intra-hospital mortality.

## Materials and Methods

### Patient enrollment

The subjects included in this analysis were male and female of 65+ years old; a sub-cohort belonging to a large case–control study in which 351 patients were eligible with final purpose to evaluate POD risk factors. Subjects were admitted at St. Orsola-Malpighi Hospital (Bologna, Italy) for any kind of emergency or elective surgery; inclusion and exclusion criteria were previously described (14) and here briefly reported. Inclusion criteria were patients of either sex above the age of 65 years, admitted and operated on the unit for any kind of emergency or elective surgery. Exclusion criteria were patients unable to perform cognitive and psychometric tests for any reason, including sensory impairment, disorders of language, and previous diagnosis of dementia. The study protocol (risk factor evaluation in post-operative delirium; RIFE POD) has been described previously (13). Ethical approval for the study was obtained from St. Orsola-Malpighi Hospital Ethical Committee (Bologna, Italy) and all procedures were followed in accordance with the Declaration of Helsinki. The presence and severity of POD among patients enrolled in the study was evaluated through confusion assessment method (CAM: sensitivity of 94–100%, specificity of 90–95% with high inter-observer agreement) and delirium rating scale (DRS: sensitivity of 82% and specificity of 94%, cut-off score of 10) interviews on post-operative days 1, 2, 3, and 6. On days 4 and 5 after surgery, CAM and DRS were administered only if hospital personnel or relatives indicated a change in patient behavior, which warranted further investigation. Both instruments are standardized tools, widely used for delirium recognition and severity assessment according to diagnostic and statistical manual of mental disorders (DSM-IV) (15). Forty-seven out of 351 patients developed POD as previously reported (14).

### Measurements of plasma cytokines level

A bio-bank of pre-operative plasma was set up for all the enrolled patients and 37 out of 47 samples from POD patients were used in the present work since these samples had never thawed. Similarly, never-thawed samples from patients who did not develop POD were randomly selected from the database and two variables, i.e., gender and age, were tested to be not significant (age–gender-matched). Pre-operative plasma cytokines levels from 74 patients were finally evaluated: 37 without POD and 37 with POD.

Venous blood was withdrawn preoperatively in the morning of hospital admission day. Plasma was obtained with a centrifugation for 15 min at 1,780 × *g* and −4°C; 300 μl aliquots were stored at −80°C until determination. Cytokine concentrations (TNF-α, IL-1β, IL-2, IL-6, IL-8, and IL-10) were measured in duplicate by multiplex sandwich ELISA technology (Human Cytokine Array 1, SearchLight, Aushon Biosystems, Billerica, MA, USA) according to the manufacturer’s instructions. Final data were obtained in a blind set up by the operators. An inflammatory score (IS) was applied, i.e., the sum of the plasma concentration of pro-inflammatory cytokines (IL-2 + IL-6 + IL-8) divided by anti-inflammatory cytokines (IL-10), as previously reported by other authors (16). Associations of cytokine levels with functional status, cognitive decline, intra-hospital mortality, and comorbidity were also analyzed independently of POD onset.

### Statistical analysis

Continuous variables were expressed as mean ± SD or ±SEM and categorical variables as percentage. Student unpaired *t*-test for continuous variables and Mantel Heantzel Chi-square test for categorical values were used. Significant variables (*p* < 0.05), identified by univariate analysis, were further examined using multivariate analysis, i.e., logistic regression, in order to evaluate potential independent risk factor for POD. To assess the association between the patient’s characteristics and cytokines level, variables were dichotomized in two groups and the differences of cytokines levels between the two groups were analyzed with Mann–Whitney *U* test. Statistical analyses were performed using SPSS^®^ software, version 15.0 (SPSS 15.0 Inc., Chicago, IL, USA).

## Results

In this study, we assessed the pre-operative plasma level of 6 cytokines (IL-1β, IL-2, IL-6, IL-8, IL-10, and TNF-α) in 74 patients: 37 POD patients and 37 patients without POD, matched for age and gender. Baseline characteristics of the two groups (POD and no-POD) are reported in Table 1 and Table S1 in Supplementary Material shows the surgical procedures in which 74 patients underwent. POD patients showed significantly higher medical comorbidities, hospital anxiety and depression, and functional decline compared to patients without POD. Pre-operative assumptions of benzodiazepines and nitrate-containing drugs were also higher in patients with POD compared to those without POD. These findings confirmed that the sub-cohort of patients we analyzed was similar to the previous one. On the other side, some variables, like hyperglycemia and SPMSQ, did not result significantly different between the two groups, likely because of the smaller sample size of the subpopulations here analyzed (14). Likewise, patients who underwent emergency surgery were not different comparing POD and no-POD (*p* = 0.601) groups.

**Table 1:** **Baseline characteristics of patients in relation to post-operative delirium (POD) onset**.

	POD (*n* = 37)	No-POD (*n* = 37)	*p*-Value
Age, mean ± SD	79.2 ± 6.7	76.4 ± 6.7	0.062
Sex ratio (M:F)	20:17	17:20	0.441
CIRS, mean ± SD	**10.3 ± 5.3**	**6.8 ± 3.6**	**0.002**
SPMSQ, mean ± SD	1.2 ± 1.4	1.1 ± 2.0	0.896
ADL, mean ± SD	**4.4 ± 2**	**5.57 ± 1.2**	**0.003**
HADS, mean ± SD	**17.6 ± 6.6**	**14.2 ± 6.1**	**0.024**
IADL, mean ± SD	**5.1 ± 2.3**	**6.6 ± 2.2**	**0.006**
Emergency surgery, *n* (%)	28 (75.7)	26 (70.3)	0.601
Nitrate-containing drugs, *n* (%)	**10 (27.0)**	**3 (8.1)**	**0.032**
Benzodiazepines, *n* (%)	**10 (27.0)**	**2 (5.4)**	**0.012**
Other hypnotics, *n* (%)	1 (2.7)	0 (0)	0.345
Antidepressants, *n* (%)	4 (10.8)	1 (2.7)	0.165
Alcohol abuse, *n* (%)	0 (0)	0 (0)	n.a.
Creatinine (mg/dL), mean ± SD	1.7 ± 1.7	1.1 ± 0.4	0.06
Blood glucose (mg/dL), mean ± SD	140.0 ± 77.0	117.3 ± 50.0	0.137
Albumin (g/dL), mean ± SD	3.1 ± 0.8	4.8 ± 8.8	0.266
Opiate, *n* (%)	1 (2.7)	0 (0)	0.345

*POD, post-operative delirium; CIRS, cumulative illness rating scale; SPMSQ, short-portable mental status questionnaire; HADS, hospital anxiety and depression scale; ADL, activities of daily living; IADL, instrumental activities of daily living; n.a., not assessable*.

**p*-Values were by student unpaired *t*-test*.

**p* ≤ 0.05 was considered significant (bold characters are reported)*.

As far as cytokines plasma level is concerned, only samples with values above the detection limit were considered and more details are reported in the Table S2 in Supplementary Material. IL-1β and TNF-α, being in the most of cases under the detection limit, were not further considered.

Baseline plasma levels of IL-6 and IL-2 were significantly different between the two groups. IL-6 was higher in POD compared to no-POD patients (*p* = 0.021), while IL-2 was significantly lower in POD with respect to no-POD patients (*p* = 0.010). We also observed lower levels of IL-10 and higher levels of IL-8 in POD compared to control subjects, but these differences were not significant (Table 2). An IS (16) showed that the two groups’ results were significantly different, as reported in Table 2, as POD group much more inflamed than no-POD group.

**Table 2:** **Plasma cytokines in patients with post-operative delirium (POD) vs. patients with no-POD**.

Cytokine pg/mL	POD		No-POD		*p*-Value
	*n*	Median (25th–75th)	*n*	Median (25th–75th)	
IL-2	35	2.50 (1.40–4.10)	33	4.80 (2.90–6.45)	**0.009**
IL-6	37	9.00 (2.25–23.65)	34	3.40 (1.85–8.95)	**0.021**
IL-8	34	6.15 (2.50–23.05)	36	5.00 (2.325–7.75)	0.245
IL-10	29	0.70 (0.25–4.00)	33	0.90 (0.50–1.85)	0.714
I score	27	45.00 (18.5–84.00)	32	18.50 (11.05–28.70)	**0.040**

*I, inflammatory score accordingly to Cerejeira et al. (16); *p*-values were calculated using Mann–Whitney *U* test for non-parametric data. Significant values are reported in bold characters*.

As the number of emergency cases was greater in both POD (28 out of 37) and no-POD (26 out of 37) groups, we performed further analysis excluding elective cases to check whether the cytokines level of the two groups were still significantly different. Both IL-2 and IL-6 persisted to be significant between the groups, *p* = 0.041 and *p* = 0.018, respectively (data not shown).

Subsequently, plasma levels of IL-6 and IL-2 were adjusted in multivariate analysis for other potential confounders, i.e., age, CIRS (comorbidity), ADL and IADL (functional abilities, activities of daily living, instrumental activities of daily living), HADS (hospital anxiety and depression), and pre-operative benzodiazepines intake. After adjustment for the above mentioned baseline covariates, the association of IL-2 with POD was lost [odds ratio 0.83 (0.63–1.08), *p* = 0.163] while the association of IL-6 persisted to be significant [odds ratio 1.1 (1.00–1.20), *p* = 0.040]. A significant IL-6 cut off between the two groups POD vs. NO-POD was ≥9 pg/mL (odds ratio between the two groups: 4.9; 95% of confidence: 1.6–14.63; *p* < 0.0005).

Independently of POD onset, high-plasma level of IL-6 was also associated with poor functional status or ADL <6 (*p* = 0.030), with cognitive decline or SPMSQ ≤7 (*p* = 0.014), emergency admission (*p* = 0.023), and higher comorbidity or CIRS ≥8 (*p* = 0.046). High-plasma level of IL-8 was significantly associated with high comorbidity (*p* = 0.009), with poor functional status (*p* = 0.048) and age <75 years (*p* = 0.022). On the other hand, low levels of IL-2 were associated with emergency admission (*p* = 0.008), higher comorbidity (*p* = 0.050), and low level of IL-10 was found to be associated with age ≥75 years (*p* = 0.045). These data are shown in Table 3. Intra-hospital mortality was also evaluated and high levels of IL-6 (*p* = 0.001) and IL-8 (*p* = 0.001) were found significantly associated with high intra-hospital mortality, as shown in Table 3.

**Table 3:** **Correlation between cytokines plasma level and different features independently of POD onset**.

	Cytokines plasma level (pg/mL)											
	IL-2			IL-6			IL-8			IL-10		
	*N*	Mean ± SEM	*p*-Value	*N*	Mean ± SEM	*p*-Value	*N*	Mean ± SEM	*p*-Value	*N*	Mean ± SEM	*p*-Value
**INTRA-HOSPITAL MORTALITY**												
Alive	60	5.9 : 1.0	0.650	64	9.6 : 3.7	**0.001**	62	8.9 : 2.3	**0.001**	54	4.2 : 0.3	0.278
Deceased	8	5.4 : 1.1		7	30.4 : 2.1		8	28.7 : 2.2		8	2.6 : 1.8	
**CIRS**												
<8	32	7.8 : 2.1	**0.027**	34	7.6 : 1.9	**0.017**	34	6.1 : 1.1	**0.022**	29	4.8 : 3.1	0.713
≥8	36	3.8 : 0.6		37	16.0 : 3.3		36	17.2 : 3.5		33	3.6 : 1.7	
**Age**												
<75 years	20	7.9 : 3.1	0.962	21	13.5 : 4.9	0.588	22	16.6 : 4.8	0.255	20	9.3 : 5.1	0.242
≥75 years	48	4.7 : 0.7		50	11.4 : 1.9		48	9.6 : 1.8		42	1.8 : 0.4	
**SPMSQ**												
>7	57	5.8 : 1.2	0.887	61	9.4 : 1.8	**0.007**	60	10.7 : 2.1	0.103	51	4.7 : 1.1	0.787
≤7	11	4.8 : 1.4		10	27.7 : 7.0		10	18.4 : 6.4		11	1.7 : 0.5	
**ADL**												
=6	44	6.9 : 1.6	0.138	47	9.8 : 2.3	**0.030**	47	10.5 : 2.5	0.620	44	5.1 : 2.4	0.763
<6	24	3.3 : 0.4		24	16.3 : 3.6		23	14.6 : 3.1		22	1.8 : 0.5	
**ADMISSION**												
Elective	17	11.7 : 3.7	**0.008**	20	9.1 : 3.6	**0.026**	18	10.8 : 4.4	0.408	15	5.3 : 3.7	0.799
Emergency	51	3.9 : 0.4		51	13.1 : 2.4		52	12.2 : 2.2		47	3.8 : 1.9	

*Values are reported as mean ± SEM; CIRS, cumulative illness rating scale; CIRS ≥8 associated with comorbidity; SPMSQ, short-portable mental status questionnaire; ≥3 associated with cognitive decline; ADL, activities of daily; ADL <6 associated with functional decline; *N*, number of patients*.

**p*-Values were calculated using Mann–Whitney *U* test. Significant values are reported in bold characters*.

## Discussion

In the present study, we observed increased levels of pro-inflammatory cytokines (IL-6 and IL-8) in POD patients, while T cell growth-related and anti-inflammatory cytokines (IL-2 and IL-10, respectively) were decreased in POD patients. The adopted IS (16) confirmed the higher level of inflammation in the POD group. After adjusting for potential confounders, pre-operative level of IL-6 was confirmed to be significantly associated with POD, thus, high-IL-6 plasma level, specifically ≥9 pg/mL (using ELISA multiplex technique), is proposed to be an independent risk factor of POD onset. We recognize that a limit of this study was the absence of information related to the use of pre-operative anti-inflammatory drugs by patients.

Few studies on POD occurrence in older patients investigated baseline inflammatory markers that can have a predictive value. Lemstra and co-workers did not find any association between pre-operative IL-6 and the incidence of delirium after hip surgery (17). However, this study was carried out in a small number of post-operative patients. Conversely, an association of IL-6 and IL-8 cytokines with delirium was found in two different works, focused on medical (18) and surgical (19) patients. In the former, authors found that IL-6 and IL-8 levels were below the detection limit in most of control cases when compared with POD patients. In the latter, van Muster and co-authors, observed highest levels of IL-6 during delirium, whereas highest levels of IL-8 were found in the days preceding delirium, thus, suggesting that a different timing of reaching the highest concentration of the two pro-inflammatory cytokines (20). A recent work showed the high-IL-2 level after surgery (20), but blood samples were examined only after surgery and measurements could be affected by surgery stressful event. In principle, only pre-operative markers can be useful to predict delirium onset and to deal with it properly.

There is a growing evidence that circulating levels of cytokines may influence the CNS (21). To this regard, a study showed higher IL-6 immunoreactivity in the brain of patients with delirium, suggesting that an association between human brain activity of microglia, astrocytes, and IL-6 and delirium in old patients (22). Interestingly, a decreased level of IL-6 was found in pre-operative cerebrospinal fluid (CSF) from elderly hip fracture patients (23), thus, suggesting not only a potential role of the molecular up-taking but also the importance of the anti-inflammatory balance in the brain. These data also suggest that two microenvironments, i.e., CSF and peripheral blood could have a different or opposite concentration of cytokines.

Currently, it is recognized that human aging is characterized by a chronic, low-grade inflammation, or “inflammaging” and IL-6, among other cytokines (24, 25), is a key mediator of this status as recently revised (26). In this work, we found that IL-6 is an independent risk factor of POD onset, thus, making more robust the theory of inflammation as crucial substrate of the great majority of age-related pathologies (cancer, osteoarthritis, neurodegeneration including Alzheimer disease and dementia, type II diabetes among other), syndromes (frailty, depression disorders), and conditions, such as sarcopenia and obesity. Thus, to better analyze the role and association of pro-anti-inflammatory cytokines with other parameters, independently of POD, we found significant higher levels of IL-6 in patients with compromised functional status, higher comorbidity, cognitive decline, and emergency admission. IL-6 was also found to be positively associated with intra-hospital mortality, thus, reinforcing the relevance of this biomarker as one of the predictors of impaired cognitive function, morbidity, and mortality (27–29).

Likewise, high level of IL-8 was also associated with comorbidity and intra-hospital mortality. Increased concentrations of pro-inflammatory cytokines are believed to contribute to cognitive decline and to predict frailty and mortality (30, 31). Moreover, low levels of IL-2 were associated with comorbidity and emergency admission. IL-2 may have neuro-regulatory effects, such as promotion of neuronal survival, stimulation of oligodendrocyte proliferation and maturation, hypothalamic-pituitary axis stimulation, and possibly analgesic properties (32). Therefore, IL-2 deficiency might favor an impairment or deregulation at brain level.

**Figure 1:**
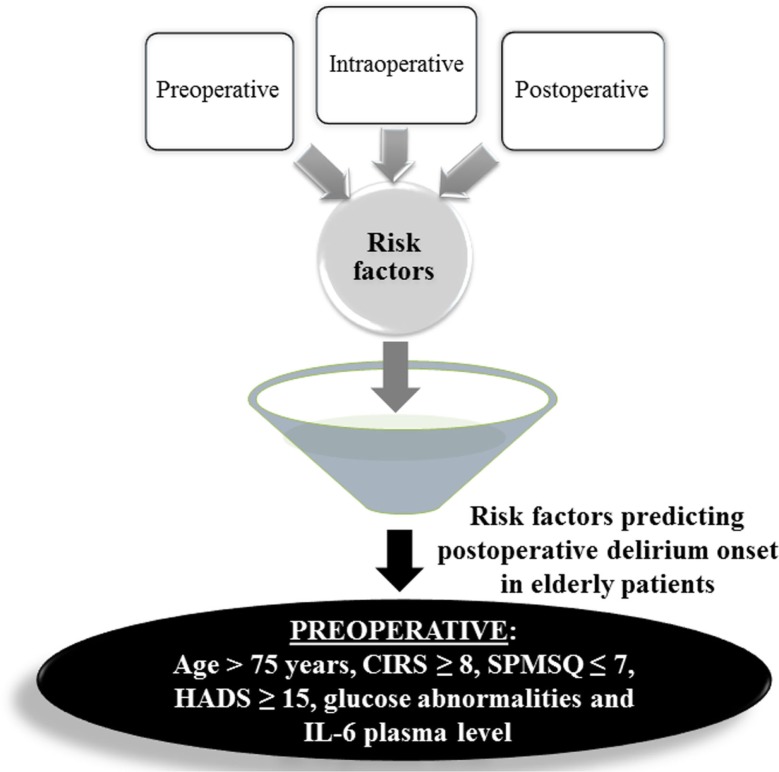
**Pre-, intra-, and post-operative risk factors were evaluated in univariate and multivariate analyses, depicted as a funnel; the numbers reported in the figure are referred to the previous work (14)**. In this paper, by means of a sub-cohort of 74 patients (37 controls and 37 with POD), pre-operative IL-6 plasma level (≥9 pg/mL) and age were found to be independent risk factors together with other pre-operative parameters, i.e., CIRS (cumulative illness rating scale), SPMSQ (short-portable mental status questionnaire), HADS (hospital anxiety and depression scale) showing that pre-operative variables are the most significant to predict POD onset.

In conclusion, the exhaustive identification of POD markers including genetic markers, as recently published (33), will allow early recognition of at risk patients. One of the most important implications of the present work is the possibility to identify subjects at high risk of POD onset at the hospitalization time taking into account the pre-operative IL-6 plasma level together with the previously identified risk factors (14), i.e., CIRS ≥8, HADS ≥15, SPMSQ ≤7, age ≥75 years, and glucose abnormalities, summarized in Figure 1. The prediction of POD onset could highly improve hospitalized patient management, and decreasing of healthcare cost. These effects were recently demonstrated with the Hospital Elder Life Program, an effective intervention to prevent delirium in older hospitalized adults, successfully replicated (34). Outcomes include a lower rate of incident delirium; shorter period of hospitalization; greater satisfaction of patients, families, and nursing staff; and significantly lower costs for the hospital. The financial return of the program, estimated at more than $7.3 million per year during 2008, comprises cost saving from delirium prevention and revenue generated from freeing up hospital beds (34). Further, the possibility to monitor longitudinally patients who had developed POD could give an advantage to the same patients to act promptly with pharmacological therapy or to counteract as much as possible the development of dementia, neurodegeneration, or Alzheimer disease with a specific lifestyle intervention.

## Author Contributions

Miriam Capri study concept and design, study management, interpretation of data, and preparation of manuscript. Stella Lukas Yani study management, analysis and acquisition of data, statistical analysis, interpretation of data, and preparation of manuscript. Rabih Chattat study concept and design, acquisition of data, and revising the article. Daniela Fortuna, Laura Bucci, and Cristina Morsiani: statistical analysis and revising the article. Catia Lanzarini acquisition of data. Fausto Catena and Luca Ansaloni study concept and design, recruitment of participants, and revising the article. Marco Adversi recruitment of patients and revising the article. Maria Rita Melotti and Gianfranco Di Nino study concept and design and revising the article. Claudio Franceschi study concept and design, interpretation of data, and revising the article. All authors have approved the submitted version.

## Conflict of Interest Statement

The authors declare that the research was conducted in the absence of any commercial or financial relationships that could be construed as a potential conflict of interest.

## Supplementary Material

The Supplementary Material for this article can be found online at http://www.frontiersin.org/Journal/10.3389/fendo.2014.00173/abstract

## Abbreviations

CIRS, cumulative illness rating scale; HADS, hospital anxiety depression scale; SPMSQ, short portable mental state questionnaire; CAM, confusion assessment method; DRS, delirium rating scale; ADL, activities of daily living; IADL, instrumental activities of daily living.
