# Supplementary material for: Pre-Operative, High-IL-6 Blood Level is a Risk Factor of Post-Operative Delirium Onset in Old Patients
Source: Front Endocrinol (Lausanne). 2014 Oct 17;5:173. doi: 10.3389/fendo.2014.00173 (PMC4201145; doi:10.3389/fendo.2014.00173)
Supplement: Supplementary file 1 [file Table1.PDF]

Table 1S. Surgical procedures performed in the 74 patients included in the study

	**POD** (n = 37)	**No POD** (n = 37)	**Total** (n = 74)
Abdominal wall, hernioplasty and adhesiolysis, n (%)	5 (13.5)	8 (21.6)	13 (17.6)
Gastric resection and gastrointestinal perforation closure, n (%)	3 (8.1)	1 (2.7)	4 (5.4)
Intestinal resection, n (%)	16 (43.2)	13 (35.1)	29 (39.2)
Cholecystectomy, appendicectomy and splenectomy, n (%)	6 (16.2)	5 (13.5)	11 (14.9)
Explorative laparotomy, stoma construction or closure, and other surgical intestinal procedures, n (%)	5 (13.5)	6 (16.2)	11 (14.9)
Salpingovariectomy, quadrantectomy, mastectomy, axillary lymph node dissection and thyroidectomy n (%)	1 (2.7)	3 (8.2)	4 (5.4)
Chest drain insertion, embolectomy, soft tissue incision and tissue incision and drainage, and limb amputation, n (%)	0 (0)	3 (8.2)	3 (4.1)

POD = postoperative delirium
