# Supplementary material for: Pre-Operative, High-IL-6 Blood Level is a Risk Factor of Post-Operative Delirium Onset in Old Patients
Source: Front Endocrinol (Lausanne). 2014 Oct 17;5:173. doi: 10.3389/fendo.2014.00173 (PMC4201145; doi:10.3389/fendo.2014.00173)
Supplement: Supplementary file 2 [file Table2.PDF]

Table 2S . Number of plasma samples in which cytokine level was detected out of 74 total samples.

IL-2	68
IL-6	71
IL-8	70
IL-10	62
IL-1β	35
TNF-α	10

Note: Due to the high number of undetectable values of IL-1β (39 of out 74 samples) and TNF-α (64 of out 74 samples) we assigned a value of 1 for detectable samples and a value of 0 for undetectable samples. No significant differences of both IL-1β and TNF-α were observed between POD and no POD groups (data not shown)
